# Bigger is better! Hippocampal volume and declarative memory performance in healthy young men

**DOI:** 10.1007/s00429-012-0497-z

**Published:** 2012-12-27

**Authors:** Sebastian T. Pohlack, Patric Meyer, Raffaele Cacciaglia, Claudia Liebscher, Stephanie Ridder, Herta Flor

**Affiliations:** Department of Cognitive and Clinical Neuroscience, Central Institute of Mental Health, Medical Faculty Mannheim, Heidelberg University, Square J 5, 68159 Mannheim, Germany

**Keywords:** Hippocampal volume, CVLT, Declarative memory, Recognition memory, Consolidation

## Abstract

The importance of the hippocampus for declarative memory processes is firmly established. Nevertheless, the issue of a correlation between declarative memory performance and hippocampal volume in healthy subjects still remains controversial. The aim of the present study was to investigate this relationship in more detail. For this purpose, 50 healthy young male participants performed the California Verbal Learning Test. Hippocampal volume was assessed by manual segmentation of high-resolution 3D magnetic resonance images. We found a significant positive correlation between putatively hippocampus-dependent memory measures like short-delay retention, long-delay retention and discriminability and percent hippocampal volume. No significant correlation with measures related to executive processes was found. In addition, percent amygdala volume was not related to any of these measures. Our data advance previous findings reported in studies of brain-damaged individuals in a large and homogeneous young healthy sample and are important for theories on the neural basis of episodic memory.

## Introduction

The hippocampus is a brain structure that has been studied extensively for its prominent role in memory and cognition. The human hippocampus is located in the medial temporal lobe, lying on the floor of the inferior horn of the lateral ventricle. The hippocampus proper is composed of the cornu ammonis (CA) subfields, whereas the hippocampal formation includes the dentate gyrus (DG), CA subfields and subiculum. All hippocampal components are composed of simple three-layered allocortex, which differentiates them from surrounding six-layered medial temporal neocortex, the entorhinal, perirhinal and parahippocampal cortices (Witter et al. 1989). The major projection to the hippocampus, the perforant path, originates in the surrounding entorhinal cortex. The entorhinal cortex and other components of the medial temporal cortex, namely perirhinal and parahippocampal cortices, are reciprocally connected with widespread areas of neocortex, predominantly association cortex (Kosel et al. 1982). Hippocampal connectivity is therefore particularly suited to a mnemonic role in that it receives information not only about the external world through sensory input but also interoceptive information about the internal state of the organism from subcortical and brainstem systems. By binding other, associative or contextual information together with the item representation supplied by the medial temporal cortices, the hippocampus provides both the necessary and sufficient conditions for the formation of episodic memories.

This anatomical evidence also provides suggestions about *how* information is encoded and retrieved during memory processing. During encoding, representations of distinct items are formed in the medial temporal cortices. These representations allow subsequent judgments of familiarity. The hippocampus then associates items and their context representations (formed in the parahippocampal cortex). When an item is subsequently presented as a memory cue, the hippocampus completes the full pattern and mediates a recovery of the contextual representation and specific item associates. The recovery of context and item associations constitutes the experience of recollection (Eichenbaum et al. 2007; Suzuki and Amaral 2004; Diana et al. 2007).

Evidence for a significant role of the hippocampus in memory processes in humans stems from the early observation of intense impairments of memory function in patients, who underwent bilateral removal of the hippocampus (Scoville and Milner 1957; Milner 1972) or the examination of patients with acquired or developmental amnesia (Yonelinas et al. 2002; Mayes et al. 2002; Vargha-Khadem et al. 1997). However, it is often difficult to assess the precise locus and extent of damage in such patients and to ascertain that only the hippocampus and no other medial temporal regions are affected. In addition, pathological heterogeneity and compensatory processes due to functional and structural plasticity complicate the determination of distinct structure–function relationships.

More recent functional imaging studies in healthy subjects also support the notion of a specific role of the hippocampus for declarative memory processes. For instance, using event-related functional magnetic resonance imaging (fMRI) Eldridge et al. (2000) demonstrated that activity in the hippocampus increased only when memory retrieval was accompanied by conscious recollection of the learning episode. However, functional imaging studies do not test whether a given region is truly necessary for the processes under investigation. Important convergent evidence about the functional relevance of a structure with regard to normal or impaired behavior can be provided by studies on the relationship between differences of this structure and inter-individual differences in cognitive performance (Kanai and Rees 2011). By this, research on individual differences in brain structure supplies information that is complementary to that provided by fMRI, since structural MRI analyses focus on the variability that gives rise to inter-individual differences in behavior, whereas fMRI studies usually reveal the most reliable activation of brain regions across individuals. These inter-individual differences in behavior, normally discarded by averaging data across participants, can be a valuable source of information and can be exploited to reveal the neural basis of distinct cognitive operations.

So far, several studies have investigated hippocampal volume in populations which are thought to have at least subtle hippocampal damage due to the influence of stress hormones or degenerative processes like patients with posttraumatic stress disorder, borderline personality disorder, childhood seizures, depression, risk of schizophrenia, an ApoE-4 allele, high estrogen levels, mild cognitive impairment and Alzheimer’s disease (Bremner et al. 1995; Cohen et al. 2001; den Heijer et al. 2003; Driessen et al. 2000; Fennema-Notestine et al. 2002; Lawson et al. 2000; O’Driscoll et al. 2001; Plassman et al. 1997; Seidman et al. 2002; Sheline et al. 1999; Simpson et al. 2001; Stein et al. 1997; VanLandingham et al. 1998; Chetelat and Baron 2003; Kantarci et al. 2002; Wolf et al. 2003). However, only few have included measures of memory performance in addition to volumetric measures. These investigations revealed positive correlations between hippocampal volume and memory only when pathology was present as in demented and amnesic patients of mixed etiologies (Barber et al. 2001; Cahn et al. 1998; de Toledo-Morrell et al. 2000; Deweer et al. 1995; Jernigan et al. 2001; Kohler et al. 1998; Kopelman et al. 2001; Mizuno et al. 2000; Mungas et al. 2002; Petersen et al. 2000) and in subjects with mild cognitive impairment (Jack et al. 2000; Soininen et al. 1994).

Only few studies investigated the relationship between hippocampal volume and declarative memory performance in healthy young subjects. In contrast to what would be expected, some of these studies reported a negative correlation between hippocampal volume and declarative memory performance (Chantome et al. 1999; Foster et al. 1999; Pruessner et al. 2007). Foster et al. (1999) argued that in healthy young people, the observed negative correlation between hippocampal volume and memory might be explained by the degree of neural pruning that has taken place during childhood and adolescence—with an inadequately pruned hippocampus mediating memory processes less efficiently than a well-pruned hippocampus. However, a recent study by Ashtari et al. (2011) showed that in a small sample of participants larger hippocampal volumes were correlated with higher verbal learning and memory scores. In addition, Rajah et al. (2010) revealed that both spatial and temporal context retrieval performance could be predicted by anterior hippocampus volume in healthy young adults. Poppenk and Moscovitch (2011) could show that recollection memory performance which is also thought to reflect retention of contextual information was positively correlated with in this case posterior hippocampal volume. A recent study in our own laboratory (Pohlack et al. 2011) used a differential fear conditioning paradigm with contextual stimuli and showed that only those persons with larger hippocampal volume learned to discriminate between two conditioned contexts, while those with small hippocampal volumes failed to do so, as indicated by skin conductance responses. However, no significant correlations between hippocampal volume measures of verbal memory performance were obtained in this study.

A meta-analysis on the mixed pattern of correlations between hippocampal volume and memory performance across the lifespan confirmed the observation of a negative relationship between hippocampal volume and memory in young adults (also in children and adolescents) (Van Petten 2004). In contrast, a positive association between hippocampal volume and memory performance was consistently found only in older samples. However, it has to be kept in mind that a variety of factors, such as the age range of the investigated participants, the validity of the various memory tests applied and the proportion of male and female participants largely differed in these examinations. By the particular task, analysis structure and participant sample employed here, the current approach aims to control as many of the variables leading to the discrepancies of prior research as possible. In the following, these sources of variance will be addressed sequentially.

One potential problem with the mixed results of those studies might be that the memory tests were not always optimal to represent hippocampal processes. Although many of the tests used across the different studies show impairments in patients with obvious hippocampal damage (Cahn et al. 1998; de Toledo-Morrell et al. 2000; Gur et al. 1999; Kohler et al. 1998; Laakso et al. 2000; O’Driscoll et al. 2001; Sanfilipo et al. 2002; Seidman et al. 2002) they might not be sensitive enough in young healthy subjects. Context memory tasks like those used in the Rajah et al. (2010), Poppenk and Moscovitch (2011) and Pohlack et al. (2011) studies appear to be much better suited to engage hippocampal processes than simpler item recognition or (cued) recall tasks (see Davachi 2006 or Giovanello et al. 2009, for fMRI evidence underpinning this assumption). Nevertheless, it has been suggested that irrespective of the exact memory test delayed tests should show a stronger relationship to hippocampal volume than those in which retrieval follows immediately after the study phase (Kohler et al. 1998). In the first published study of hippocampal volume–memory relationships in a non-demented sample of older adults, Golomb et al. (1994) observed a significant correlation between hippocampal volume and delayed, but not immediate recall. However, the total performance on delayed recall is not independent from what has been learned previously, since delayed recall confounds retrieval with acquisition. Consequently, by taking into consideration only the pure number of retrieved items after distraction or delay one runs the risk of contaminating hippocampal dependent memory processes with extra-hippocampal processes during acquisition. A low delayed memory score can thus result from a loss of information over the delay period *or* from a low learning rate during encoding. The first process would be an indicator of impaired hippocampal functioning while the latter can also result from impaired prefrontal cortex mediated executive or working memory processes (Helmstaedter et al. 1997). Therefore, it is preferable to use a quotient which relates the loss in delayed recall to the number of words learn. Moreover, to delineate functional specificity, it is important to use memory tests in which some scores presumably rely on hippocampal processes while others do not and to include a control brain region, which should not be associated with declarative long-term memory processes. Most tests of verbal memory only yield an overall measure of performance which does not allow determination and differentiation of the various cognitive subprocesses of declarative memory. This is problematic as the same overall score can be achieved for different reasons. To overcome this problem, the German version of the California Verbal Learning Test (CVLT) (Delis et al. 1987; Niemann et al. 2008) was used in this study. The various components of the CVLT permit a clearer separation and a more fine-grained evaluation of critical memory subprocesses and can, therefore, provide more specific information about structure–function relationships. In previous research, the CVLT has already been associated with hippocampal activation (Johnson et al. 2001) and hippocampal volume in posttraumatic stress disorder (Tischler et al. 2006) as well as in Alzheimer’s disease (Walhovd et al. 2004).

A further significant factor contributing to the inconsistent pattern of correlations between memory and hippocampal volume in healthy young subjects in the existing literature is likely to be the stage of menstrual phase in women. Animal research indicates that sex hormones can affect hippocampal plasticity (Woolley and McEwen 1992; Yankova et al. 2001; Gould et al. 2000). For instance, in a study conducted by Woolley and McEwen (1992), a 32 % decrease in hippocampal synaptic density was detectable 24 h following the onset of rat estrus. In humans, post-menopausal estrogen supplementation has been associated with greater hippocampal size (Eberling et al. 2003). A recent study (Protopopescu et al. 2008) used voxel-based morphometry to investigate regional variation in gray matter over the menstrual cycle. Each woman was scanned during the postmenstrual late-follicular phase and during the premenstrual late-luteal phase. In the right anterior hippocampus gray matter was relatively increased in the postmenstrual phase. In addition, verbal declarative memory scores were better in the postmenstrual versus premenstrual phase. Goldstein et al. (2010) reported significant sex differences in hippocampal activity which were dependent on the phase of the women’s menstrual cycle. In contrast to men, women in their late follicular–midcycle menstrual phases exhibited significantly lower hippocampal BOLD signal changes in response to stressful stimuli. To reduce heterogeneity in the current sample, we decided to include only male participants in the present study.

Regional brain volume can be conceived as a function of the number of neurons and their interconnections and as an indicator of its processing capacity (Striedter 2005; Barton 1998). Consequently, individuals with larger brain regions should perform functions mediated by those regions better—provided that the critical factors described above are appropriately controlled. Thus, differences in performance and volume can be used as a source of information to link cognition to brain anatomy. We hypothesized that percent hippocampal volume should be positively associated with selective hippocampus-dependent measures of verbal declarative memory performance like short-delay and long-delay retention as well as discriminability. As we were interested in the specificity of these associations, we also expected that other measures of presumably extra-hippocampally mediated processes like total learning or proactive interference should not be related to hippocampal size. Finally, percent amygdala volume (as a performance-unrelated control region) should not predict performance in any of the declarative memory scores.

## Materials and methods

### Subjects

Fifty healthy young male volunteers (age range 19–27 years, mean age 21.54 (SD 2.09), 47 right-handed) participated in the study. The current cohort represents a subset of participants from our previous study on the relationship between hippocampal volume and performance in context conditioning (Pohlack et al. 2011). In comparison to this, previous investigation participants were excluded if only incomplete data were available for the measures of episodic memory or hippocampal and amygdalar volumes. Furthermore, no data from female participants were analyzed. Volunteers suffering from a current mental disorder (axis I/II), including substance dependence or abuse, as assessed using the German Version of the Structured Clinical Interview for the Diagnostic and Statistical Manual of Mental Disorders-IV [SCID I (Wittchen et al. 1997)], were excluded from the study. The Ethics Committee of the Medical Faculty Mannheim of the University of Heidelberg approved the study, and we obtained written informed consent from all persons before participation.

### Declarative memory assessment

Declarative memory was assessed with the German version of the California CVLT (Delis et al. 1987; Niemann et al. 2008). In the CVLT, a list of 16 words (list A) is presented five times in succession, and subjects are instructed to recall as many words as possible after each presentation of the word list. After the five test trials of the first list (list A), a new list of words (list B) is read to the subjects, who are instructed to recall as many words as possible from list B. Subjects are then asked to recall list A again (short delay) and, after a 20-min interval, are again asked to recall list A (long delay). Finally, recognition memory is examined after the long-delay cued recall by reading aloud each word on a 44-word list, while subjects must indicate whether it is a target word (i.e., from list A) or a distractor. Distractors or “lures” are either words from list B, share semantic categories with the target words, or sound alike.

### Statistical analyses

The dependent variables were (1) number of words recalled following trials 1 through 5 (*total learning*), (2) number of words recalled from list B in relation to number of words recalled from list A after the first learning trial (proactive interference [(list B−learning trial 1)/learning trial 1 × 100]), (3) loss of learned words after a short delay (short-delay retention [(short delay free recall−learning trial 5)/learning trial 5 × 100)]), (4) loss of learned words after a longer delay (long-delay retention [(long delay free recall−learning trial 5)/learning trial 5 × 100)]), and (5) a recognition test which yields a measure of *discriminability* (Pr) to quantify the extent to which participants correctly discriminate target from non-target words [*p* (hits)−*p* (false alarms)].

Pearson’s correlation tests were used to assess the associations among percent hippocampal volume, percent amygdala volume and memory performance.^1^

To eliminate potential effects of age and intelligence [assessed by the multiple-choice vocabulary test MWT-B (Lehrl 2005)] additional partial correlations were conducted. Owing to our directional a priori hypotheses, only one-tailed tests were calculated. We controlled for effects of multiple testing by the Benjamini–Hochberg method (1995). Data from participants deviating more than two standard deviations from the mean of a specific score were removed.

### Structural magnetic resonance imaging

Data acquisition was conducted using a 1.5-T whole body MR-scanner (Magnetom VISION, Siemens Medical Solutions, Erlangen, Germany) with a head volume coil. Images for the volumetric analyses were acquired with a three-dimensional spoiled gradient (FLASH, TR 15 ms, TE 5 ms, FOV 256 × 256 mm^2^) resulting in 170 sagittal slices with a voxel size of 0.86 × 0.86 × 1 mm. For the amygdala protocol, a T2-weighted interleaved Turbo Spin Echo sequence was acquired (TE 54 ms, TR 7,280 ms, FoV 220 × 220 mm^2^, matrix 256 × 256 × 1) with the following parameters: slice thickness 2 mm, gap 2 mm, flip angle 180°, voxel size 0.86 × 0.86 × 4 mm. Afterwards, each image was resampled to a 1 mm^3^ voxel size. T2-weighted images were reoriented to optimally match the T1-weighted images. Raw images were converted to BRAINS2 (Magnotta et al. 2002) readable format with the freely available software MRIcro (Rorden and Brett 2000). Manual tracing of hippocampus and amygdala was conducted by two trained operators unaware of the neuropsychological data of the participants using BRAINS2. The two structures were outlined in native space to avoid the distortions intrinsic to normalization.

### Hippocampal volume assessment

**Fig. 1:**
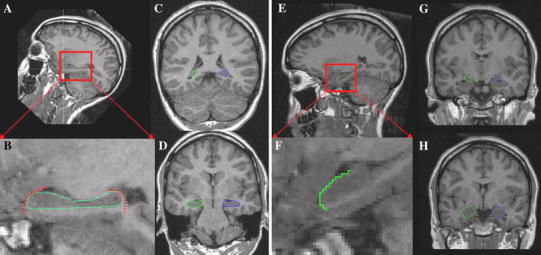
Manual segmentation of the hippocampus and the amygdala. **a** For the tracing of the hippocampus, each brain was realigned along its longitudinal axis. **b** *Magnification* of the sagittal view showing the outlined hippocampus. This step enhances segmentation accuracy in critical coronal slices. **c** Final coronal segmentation started always at the posterior end of the hippocampus. **d** Exemplary coronal slice of the hippocampal head. **e** For the amygdala tracing, the brains remained oriented along the anterior–posterior commissure (AC–PC) line as originally measured. **f** *Magnification* of the sagittal view showing how the hippocampal head was separated from the amygdala. **g** Again, segmentation was conducted from the posterior part to the anterior end of the amygdala. **h** Exemplary slice of the* left* and* right* amygdala. Details on the segmentation protocols can be found in the “Materials and methods” section

One rater (S.P.) performed the manual volumetric measurements as described in a detailed anatomical guidelines (Malykhin et al. 2007). After smoothing, images were reoriented along the longitudinal axis of the hippocampus using the resample function of BRAINS2. As a next step, outlining of the hippocampus in each relevant sagittal plane followed to improve segmentation accuracy in critical coronal slices. The resulting region of interest (ROI) was used as basis for a coronal ROI, which was outlined to assess hippocampal volume (Fig. 1). Tracing always started at the posterior end of the hippocampus. Finally, a visual inspection of the resulting three-dimensional shape was conducted using the surface interface. The first ten brains were assessed three times to establish intrarater reliability: twice in the beginning and once again after the entire sample was segmented. The intraclass correlation coefficients of 0.98 at both time points suggest a high stability. Further, a second rater (R.C.) manually assessed ten hippocampal volumes to establish interrater reliability. The intraclass correlation coefficient of 0.92 signified high reliability.

### Amygdala volume assessment

The volume of the amygdala was calculated as a control region to hippocampal volume. A trained operator (R.C.) carried out a manual tracing protocol based on the published standardized guidelines (Nacewicz et al. 2006; Pruessner et al. 2000; Szeszko et al. 2004). The coronal plane was oriented along a straight line through anterior and posterior commissure to achieve the optimal orientation for manual tracing (Brierley et al. 2002). First, using both T1- and T2-weighted images, the head of the hippocampus had to be visually separated from the ventro-caudal amygdala surface in the sagittal section. The superior border was defined in the coronal plane by a straight line drawn from the fundus of the circular sulcus of the insula to the dorsolateral aspect of the optic tract. Tracing was conducted from posterior to anterior slices (Fig. 1). After tracing, refinement of each amygdale through plane-by-plane comparison followed as well as a comparison with ex vivo atlas sections (Talairach and Tournoux 1993). As for the hippocampus, intraclass coefficients (as a measure of intrarater reliability) of 0.98 for the right and 0.99 for the left amygdala suggest high stability. Again, a second trained rater (S·P.) outlined 10 brains manually to assess interrater reliability, yielding an intraclass correlation coefficient of 0.88.

### Total brain volume measurement

Total brain volume (sum of gray and white matter) was additionally assessed to conduct head-size corrections. Based on the individual T1-weighted images, total brain volume was calculated for each participant using the brain extraction tool [BET (Smith 2002)] and the automated segmentation tool [FAST (Zhang et al. 2001)] from the FMRIB software library (http://www.fmrib.ox.ac.uk/fsl/).

## Results

**Fig. 2:**
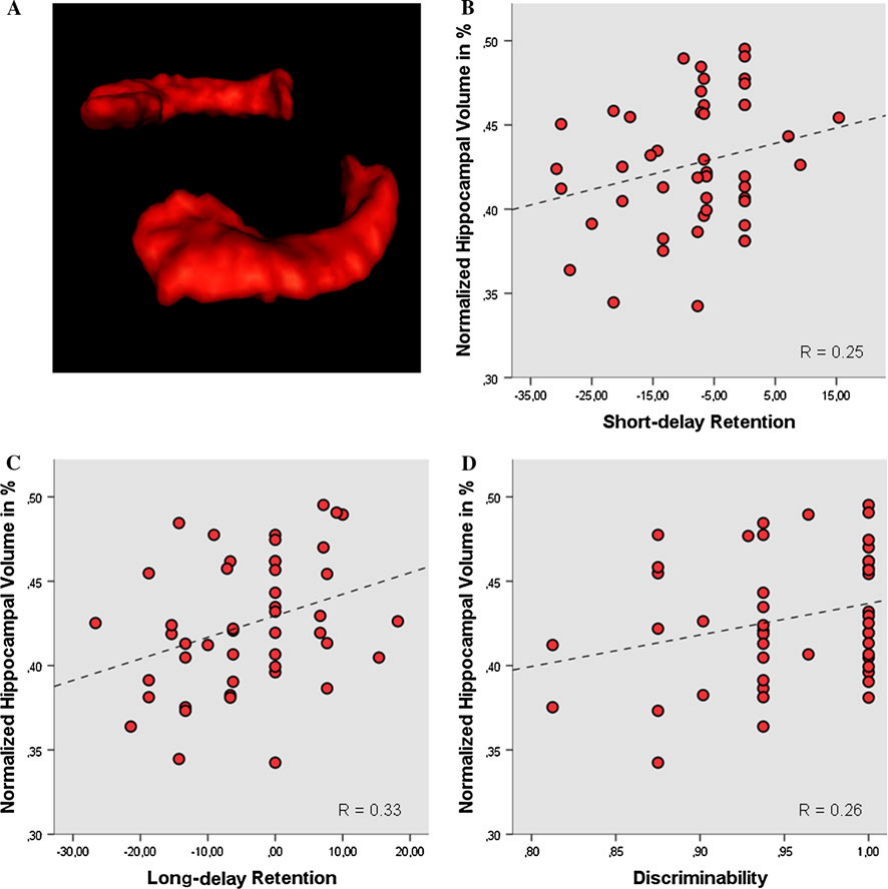
Three-dimensional rendering of the* left* and the* right* hippocampus (**a**). *Scatterplots*, including lines of best fit, showing the relationship between the normalized hippocampal volume in % and **b** short-delay retention, **c** long-delay retention, and **d** discriminability performance

The demographic data of the study are described in Table 1. Percent hippocampal volume was positively associated with *short*-*delay* *retention* (*r* = 0.25, *P* = 0.04, *n* = 48), *long*-*delay retention* (*r* = 0.33, *P* = 0.01, *n* = 47) and *discriminability* (*r* = 0.26, *P* = 0.03, *n* = 48) (Fig. 2). These results remained significant after correction for multiple testing. No significant associations were found for the measures of *total learning* (*r* = 0.06, *P* = 0.35, *n* = 48) and *proactive interference* (*r* = −0.08, *P* = 0.28, *n* = 48). In addition, no significant association could be found between percent amygdala volume and any of those measures (all *rs* < 0.17 (range 0.00–0.17); all *Ps* > 0.09 (range 0.09–0.48); *n* = 44–45).

**Table 1:** Subject demographics

	Mean	Standard error
Age	21.54	0.29
Intelligence	107.82	1.70
Total brain volume (mm^3^)	1,266.44	11.06
Normalized hippocampal volume (%)	0.42	0.005
Normalized amygdalar volume (%)	0.28	0.003
Short-delay retention	−8.39	1.49
Long-delay retention	−3.93	1.47
Discriminability	0.95	0.007
Total learning	60.85	1.14
Proactive interference	−8.74	3.65

Percent hippocampal volume was still positively associated with *short*-*delay* *retention* (*r* = 0.25, *P* = 0.04, *n* = 48), *long*-*delay retention* (*r* = 0.33, *P* = 0.01, *n* = 47) and *discriminability* (*r* = 0.29, *P* = 0.02, *n* = 48) when age and intelligence were partialled out. In addition, these results remained significant after correction for multiple testing. Again, no significant association with percent hippocampal volume was found for the measures of *total learning* (*r* = 0.05, *P* = 0.37, *n* = 48) and *proactive interference* (*r* = −0.08, *P* = 0.29, *n* = 48) with age and intelligence controlled for. Partial correlations did not reveal any significant association between percent amygdala volume and the memory measures (all *Rs* < 0.19 (range −0.19 to 0.19); all *Ps* > 0.11 (range 0.11–0.48); *n* = 44–45).

To control for effects of influential points, we repeated all analyses excluding influential observations by a statistical procedure proposed by Belsey et al. (2005). Observations with studentized residuals larger than two in absolute value were removed. These analyses revealed very similar results as those for the uncorrected sample with regard to short-delay retention (*r* = 0.29, *P* = 0.02, *n* = 44) and long-delay retention (*r* = 0.42, *P* < 0.01, *n* = 44). Only the coefficient of the discriminability measure merely survived as a trend (*r* = 0.19, *P* = 0.09, *n* = 46).

## Discussion

This study reports that in healthy young males hippocampal volume is positively associated with declarative memory performance as tested by the CVLT. Measures of *short*-*delay* *retention*, *long*-*delay* *retention* and *discriminability* were significantly positively correlated with percent hippocampal volume. This was not the case for *total learning* and *proactive interference*. In addition, percent amygdala volume was not related to any of these measures. To our knowledge, this is the first study to show a direct association between hippocampal volume and declarative memory performance in healthy young adults using standard neuropsychological testing procedures. These findings illustrate that individual differences in episodic memory function are partly driven by differences in hippocampal volume. By this, they confirm and extend previous functional studies that provided support for a crucial role of the hippocampus in episodic memory by linking structural, instead of functional, correlates to episodic memory performance.

The selective pattern of the reported correlations is in line with the previous research on those distinct declarative memory measures. *Proactive interference* effects were not significantly correlated with hippocampal volume in our study. Also in neuropsychological case studies *proactive interference* has been found to be comparable between patients with anterograde amnesia and controls (Warrington and Weiskrantz 1978). Moreover, there are convergent data which show that it is the lateral prefrontal cortex which is associated with retrieval related interference induced by previously learn stimuli, as revealed by functional neuroimaging (D’Esposito et al. 1999; Jonides et al. 1998; Wolf et al. 2010) and neuropsychological lesion studies (Thompson-Schill et al. 2002). In a study by Vanderploeg et al. (1994), relationships between executive abilities and performance on the CVLT were examined in a sample of neurological patients. Using a principal components factor analysis, they demonstrated that *proactive interference* was associated with working memory capabilities, presumably mediated by the lateral prefrontal cortex. Although, in general, their data suggest only a weak relationship between the CVLT indices and executive function variables in terms of average shared variance, the total learning consistently showed the highest correlations with measures of executive function. For that reason, comparable verbal memory tests like the VLMT (Helmstaedter et al. 1997) also take the total learning score as a measure of the short-term aspect of learning or working memory. The difference between, for instance, a story recall memory test and list learning as used in the CVLT is that in the case of list learning participants must heavily rely on abilities to organize the material for optimal learning and recall using strategic encoding and semantic clustering. As no distractions or delays occur, hippocampal processing is not essential. Consequently, individuals with frontal lobe dysfunction, who have difficulties in flexibly allocating cortical activation in response to processing demands perform poorly on the CVLT with respect to total learning (Hazlett et al. 1998). Also Brooks et al. (2006) showed that older adults with executive impairments performed significantly worse with regard to total learning than a control group while performance on other measures of the CVLT like long-delay free recall or recognition did not differ.

*Short*-*delay retention* reflects the loss of learned words after a short delay in relation to the last learning trial. Although the delay is quite short, this score clearly represents a measure of hippocampus-dependent long-term memory processes, as another word list had to be learn and recalled in between. Consequently, information cannot be retrieved from short-term memory. This measure can be also described as “retroactive interference”. Recent research has shown that both patients with amnestic mild cognitive impairment and with anterograde amnesia are severely prone to effects of retroactive interference (Dewar et al. 2009, 2010). Both conditions have been associated with impaired hippocampal functioning (Markesbery 2010; Cipolotti and Bird 2006).

The loss of learned words in delayed recall—as a measure of *long*-*delay retention—*has been found to critically rely on hippocampal integrity. Helmstaedter et al. (1997) studied verbal declarative memory in epilepsy patients being considered for surgical resection of the temporal lobe using a word list paradigm similar to the CVLT. Verbal memory was evaluated preoperatively, during the recording of intracranial event related potentials (ERPs) and postoperatively after selective hippocampectomy, temporal cortical lesionectomy, or anterior two-thirds *en bloc* temporal lobe resection procedures. Both preoperative differences in verbal memory performance as a function of differences in underlying neuropathology, intracranial ERPs, and specific patterns of postoperative memory impairments supported the notion that *long*-*delay retention* is in particular mediated by the hippocampal formation.

The association between measures of recognition memory like *discriminability* and hippocampal functioning has been intensively discussed in the literature. Although some findings suggest that both recall and recognition are impaired in patients with adult-onset apparently selective hippocampal damage (Manns and Squire 1999), others found that hippocampal damage—at least early in life—impairs recall but can spare item recognition (Vargha-Khadem et al. 1997). This second line of evidence underpins the notion that the hippocampus supports the contextual retrieval of episodic memory (required in recall tasks), whereas adjacent structures like the perirhinal cortex support the retrieval of item information, a form of retrieval that supports recognition related familiarity, which is not beneficial in recall tasks. However, the presence or absence of an association between measures of recognition memory and hippocampal functioning is completely reliant on the nature of the recognition task applied. Critically, it depends on target–lure similarity and test format. This was found in experiments comparing a focal adult-onset hippocampal amnesic (patient Y.R.) with control participants (Holdstock et al. 2002; Mayes et al. 2002). She performed poorly relative to controls on standard YES/NO recognition tests with related lures, but performed relatively well on Yes/No recognition tests that use unrelated lures. The clear impairment on Yes/No recognition tests when targets and lures are similar, is predicted by the neural network-based complementary learning systems model of recognition memory (Norman and O’Reilly 2003). This model postulates that recognition is mediated by hippocampus-dependent recollection and familiarity which relies on the cortical part of the medial temporal lobe such as the perirhinal or the entorhinal cortices; thus hippocampal damage should not impair item familiarity. However, the model also postulates that familiarity is ineffective when similar targets and lures are shown and subjects have to identify which items are old because the representations of studied items and lures overlap in medial temporal lobe cortex. In contrast, the hippocampal recall signal is more diagnostic as most lures do not trigger any recall because of the tendency of the hippocampus to assign distinct representations to stimuli regardless of their similarity. From this view, it follows that the medial temporal lobe cortex cannot support recall of details from specific events owing to its inability to sufficiently differentiate the representations of different events. This can only be achieved by hippocampal processes using separated representations to encode the details of specific events while minimizing interference—a process called pattern separation. The CVLT-recognition memory test is also a Yes/No recognition test with related lures. These are either words from list B from overlapping categories, new words that share semantic categories with the target words, or that sound alike. Thus, in terms of the findings discussed above, hippocampal processing is required to solve this task.

Work that has previously been conducted on the relationship between the volume of the hippocampus and memory performance in healthy young adults has tended to produce a somewhat equivocal pattern of findings. Except for the studies by Rajah et al. (2010), Ashtari et al. (2011) and Poppenk and Moscovitch (2011) these investigations have either observed a negative correlation between hippocampal size and memory performance or failed to find a significant association between hippocampal volume and memory performance (Van Petten 2004). However, as stated previously, these prior studies differ, in some respects, from the present investigation by either enrolling female participants without controlling for menstrual cycle phase (Chantome et al. 1999; Pohlack et al. 2011; Foster et al. 1999), small sample size (Pruessner et al. 2007; Ashtari et al. 2011), including no correction factor for overall differences in head size (Pruessner et al. 2007; Ashtari et al. 2011), using no control measures of presumably non-hippocampus dependent functions (Foster et al. 1999; Pruessner et al. 2007) or including no control brain region that was not expected to correlate with declarative memory measures (Foster et al. 1999; Chantome et al. 1999).

The present study investigated a homogeneous group of participants consisting of only male subjects in a similar age range and with similar educational background. We showed that it is the percent hippocampal volume and not a control brain region like the amygdala that is correlated with specific declarative memory measures like short-delay retention, long-delay retention and discriminability and not with others. The present findings are important for theories concerning the neural mediation of episodic memory, as well as for the significance of the volume of the hippocampus in healthy young individuals. Moreover it complements previous findings reported in studies of brain-damaged individuals (see Van Petten 2004 for a review).

This study has several limitations. Although the current data indicate a broadly linear relationship between percent hippocampal volume and critical declarative memory measures, the exact parameters of this relationship will need to be worked out in future research. Moreover, to gain a better understanding of the relationships among hippocampal volume and declarative memory performance over the life span, individuals of different ages should also be included and/or a longitudinal study should be designed. In addition, the inclusion of women whose position in the menstrual cycle is controlled for by blood assays could render the results more representative. In addition, it has to be kept in mind that the quality of manual tracing of brain structures is always restricted by the actual resolution of the MRI images—in our case the images were acquired in a 1.5-T Scanner—and that very high inter- and intra-rater reliabilities do not indicate anatomical accuracy, but only stability of segmentation.

Another limiting factor might be that no other memory related structures in the medial temporal lobe like the perirhinal, entorhinal and parahippocampal cortices have been investigated in the present study. Although the critical memory scores under investigation are hypothesized to rely mainly on hippocampal processing, a contribution of extrahippocampal medial temporal lobe structures is likely as the hippocampus proper operates on information provided by these structures (Eichenbaum et al. 2007). However, an exact delineation of these structures is difficult due to considerable variability in shape and size across individuals. In other words, the assumed hippocampus-dependent measures probably do not exclusively reflect hippocampal processing. The different memory operations expressed in these measures are suggested to be reliant on the complex interplay between various interconnected brain structures (including, e.g., also the prefrontal cortex or the parietal lobe) where the hippocampus is only one, but a very important part. As a consequence, it is not possible to completely isolate the contribution of a single memory-related brain structure with standard neuropsychological tests or with more sophisticated designs. A limitation of these data is that they cannot dissociate between prefrontal executive processes and hippocampal functions as this would require the examination of correlations between CVLT measures and the volumes of prefrontal cortex regions putatively involved in executive functions. The focus of the present investigation was to provide information about the functional relevance of the hippocampus for specific memory processes. As it is always complicated to interpret null results, our data point more to a lack of functional relevance of the hippocampus in particular memory processes like proactive interference or total learning than to the functional relevance of the prefrontal cortex in these processes.

Moreover, in the present study, we cannot determine to what extent the relationship between hippocampal volume and memory performance depends on the relative size of the anterior and posterior segments of the hippocampus. Poppenk and Moscovitch (2011) showed that better episodic memory was associated with larger posterior and smaller anterior hippocampal segments while overall hippocampal volume did not predict memory function. In contrast, Rajah et al. (2010) showed context memory performance in healthy young adults was related to anterior hippocampal volume. Moreover, there are numerous neuroimaging studies that report that the anterior hippocampus is implicated in episodic memory (Chadwick et al. 2010; Davachi 2006; Hirshhorn et al. 2012). Thus, the unresolved issue of a differential engagement of anterior versus posterior segments of the hippocampus in episodic memory tasks needs further investigation. Nevertheless, the current data suggest that the CVLT is able to assess overall hippocampal functioning.

Another open issue is the optimal method of hippocampal volume normalization. The application of multiple correction methods represents a critical source of variance in previous studies. In general, morphometric studies should include a correction factor for overall differences in head size before the relationship between the volume of any given brain structure and cognitive performance is examined. However, the exact influence of proportional measures of hippocampal volume on structure–function correlations is unclear (Van Petten 2004). Owing to the investigation of two separate brain regions in this study, we used a volume divided by total brain volume account to provide the relative contribution of both structures to memory performances. The same approach was used by Chantome et al. (1999) who only could show a (negative) relationship between hippocampal volumes and episodic memory performance when the total brain volume was taken into consideration.

A further open issue is the possibility that smaller hippocampal volumes might be compensated by increases in activity, which has previously been found in patients with mild cognitive impairment (Dickerson and Sperling 2008) or in cognitively intact carriers of the APOE-ε4 allele (Wierenga and Bondi 2007).

In an attempt to resolve the discrepancies in previous research with regard to hippocampal volume and memory performance, this study used a standard neuropsychological test procedure with subscales that can differentiate global hippocampal functioning. Moreover, to prevent influences from varying sex hormone levels and age, only male participants in a narrow age range were enrolled in this investigation. Hippocampal volume set in relation to total brain volume was positively correlated with short-delay retention, long-delay retention and discriminability. These results can help to enhance our understanding of structure–function relationships of the hippocampus and to guide further research on these factors in a variety of disorders. Finally, the present data can only show an association between the volume of a particular brain region and behavioral performance And such correlational associations do not imply causal relationships. To complement such correlational analyses, we suggest that lesion studies investigating amnesic patients with circumscribed hippocampal damage—controlling for the critical factors discussed above—should be conducted or reconsidered to provide independent support for a causal link between structure and function.

## Footnotes

1 In general, morphometric studies should include a correction factor for overall differences in head size before the relationship between the volume of any given brain structure and cognitive performance is examined. Here, we used volume divided by total brain volume as correction factor.
